# When mites attack: domestic mites are not just allergens

**DOI:** 10.1186/1756-3305-7-411

**Published:** 2014-08-29

**Authors:** Yubao Cui

**Affiliations:** Department of Laboratory Medicine, Yancheng Health Vocational & Technical College, Jiefangnan Road 263, Yancheng, Jiangsu Province 224006 P.R. China

**Keywords:** Human acariasis, Pulmonary acariasis, Intestinal acariasis, Urinary acariasis, Oral mite anaphylaxis (Pancake Syndrome), Otoacariasis, Vaginal acariasis

## Abstract

Domestic mite species found in indoor environments and in warm or tropical regions are well known for causing allergic disorders. However, little is known about human acariasis, in which mites invade and parasitize the human body in various tissues from the gastrointestinal tract to the lung. Here, we summarize the reported cases of human acariasis of pulmonary, intestinal, oral (anaphylaxis), urinary, otic, and vaginal systems. Because the clinical symptoms of acariasis often overlap with other disease symptoms leading to frequent misdiagnosis, we highlight the need for more attention on these infections.

## Background

“Domestic mites” refers to a variety of small (0.5 mm) arthropods found living in close proximity to humans (Table 1). They include house dust mites and storage mites. Typically belonging to the family Pyroglyphidae (Analgoidea, Astigmata, Acariformes), the house dust mites are commonly found in human homes. Some other mites can be present in house dust, especially several species from the families Glycyphagidae, Aeroglyphidae Chortoglyphidae, Echimyopodidae, Tyroglyphidae, Lardoglyphidae, and Suidasiidae, as well as species of the families Tarsonemidae and Cheyletidae. The latter are typically regarded as storage mites because they occur widely in-and contribute to the deterioration in quality of—stored products like grains, foodstuffs, and Chinese medicinal herbs [1, 2]. They are also found in mattresses, pillows, and upholstery. Both the pyroglyphid mites and other mite species that are found in house dust can provoke an IgE antibody response in people worldwide [3–16]. These mites have been widely studied because their feces, eggs, and extracts from their bodies are potent allergens. Indeed, approximately 10% of the total population and 90% of asthmatic patients are allergic to domestic mites [17].

**Table 1:** **The taxonomy of domestic mites and common species**

Common names	Super family	Family	Genus	Species
**House dust mites**	Analgoidea			
		Pyroglyphidae		
			*Dermatophagoides*	*D. pteronyssinus*
				*D. farinae*
				*D. microceras*
				*D. siboney*
				*D. evansi*
				*D. neotropicalis*
			*Hirstia*	*H. chelidonis*
				*H. domicola*
			*Sturnophagoides*	*S. bakeri*
			*Malayoglyphus*	*M.intermedius*
				*M.carmelitus*
			*Euroglyphus*	*E. maynei*
			*Gymnoglyphus*	*G.longior*
			*Hughesiella*	*H. africana*
**Storage mites**	Glycyphagoidea			
		Chortoglyphidae	*Chortoglyphus*	*C. arcuatus*
		Echimyopodidae	*Blomia*	*B. tropicalis*
		Aeroglyphidae	*Glycycometus*	*G. malaysiensis*
		Glycyphagidae	*Gohieria*	*G. fuscus*
			*Glycyphagus*	*G. domesticus*
			*Lepidoglyphus*	*L. destructor*
	Acaroidea			
		Tyroglyphidae	*Acarus*	*A. siro*
			*Tyrophagus*	*T. putrescentiae*
			*Aleuroglyphus*	*A. ovatus*
		Suidasiidae	*Suidasia*	*S. nesbitti*
				*S. medanensis*
		Lardoglyphidae	*Lardoglyphus*	*L. zacheri*
				*L. konoi*

Although domestic mites are well known allergens, they are also responsible for other, non-allergic, symptoms in humans, called acariasis. Little is known about acariasis, in which mites invade and parasitize the human body in various tissues from the gastrointestinal tract to the lung. Mites, throughout their lives or during individual life cycle stages, commonly parasitize animals, and some free-living mites can invade a host only occasionally. When domestic mites invade a human body, it is believed to be a non-specific invasion differing from parasitization of an animal, because the non-specific invasion occurs only occasionally and lacks specific symptoms and are not caused by specific mite species; in contrast, specific invasions are caused by specific parasite species occurring in specific hosts and producing specific symptoms [18]. To date, few reports of human acariasis have been published in English medical journals. However, acariasis has been described for pulmonary, intestinal, oral (anaphylaxis), urinary, otic, and vaginal systems. Importantly, the clinical symptoms of acariasis are non-specific and often overlap with other disease symptoms, leading to frequent misdiagnosis and, likely, many missed cases. In this review, we present the types of acariasis that have been described, and summarize the current knowledge about these little known and little-understood infestations (Table 2).

**Table 2:** **A simple summary of reported cases of human acariasis**

Acariasis types	Location (s)	Mite species	Symptoms	Diagnosis	Treatment	Refs
Pulmonary	E. Africa, Korea, China	*Acarus siro, Tyrophagus putrescentiae, Aeuroglyphus ovatus, Caloglyphus berlesei, Thyreophagus entomophagus, Suidasia nesbitti, Dermatophagoides farinae, D. pteronyssnius, Euroglyphus maynei, Tarsonemus granarius, Tarsonemus floricolus Chortoglyphus arcuatus, Cheyletus malaccensis. Cheyletus eruditus, Caloglyphus mycophagus, Largoglyphus zacheri, Lardoglyphus konoi.*	Continuous dry cough, wheezing, pain in the chest, increasing dyspnea, bronchiectasis	Identifying mites in sputum	Organo-arsenic drugs like carbarsone and acetarsol, as well as drugs like hetrazan, thiodiphenyl-amine, emetine, and some antibiotics	[19–22]
Intestinal	Spain, China	*Dermatophagoides farinae*, *D. pteronyssinus*, *Acarus siro*, *Tyrophagus putrescentiae*, *Tyrophagus longior*,*Carpoglyphus domesticus*, *Glycyphagus domesticus, G.ornatus, G. privatus*, *Carpoglyphus lactis* and *Tarsonemus granarius, Suidasia* mites	Diarrhea, abdominal pain, abdominal discomfort, mucous stools, blood and pus, anal burning sensation, fatigue, weight loss, lack of energy, asthma, vomiting, loss of appetite, fever	Identifying mites in stools	ivermectin	[21–29]
Urinary	Canada, Romania China, S. Africa	*Histiogaster, Acarus siro, Tyrophagus putrescentiae, T. longior, Aleuroglyphus ovatus, Caloglyphus berlesei, C. mycophagus, Suidasia nesbitti, Lardoglyphus konoi, Glycyphagus domesticus, Carpoglyphus lactis, Lepidoglyphus destructor, Dermatophagoides farinae, D. pteronyssinus, Euroglyphus maynei, Caloglyphus hughesi, Tarsonemus granarius and T. hominis*	Frequent desire to urinate, pyelonephritis, and pyelocystitis	Identifying mites in urine	Chloro-quine, metro-nidazole	[58–63]
Oral (Anaphylaxis)	Japan, Canada, Spain	*Dermatophagoides farinae*, *Dermatophagoides pteronyssinus, Tyrophagus putrescentiae*, *Thyreophagus entomophagus, Blomia freemani, Blomia tropicalis*, *Suidasia medanensis*, *Aleuroglyphus ovatus*, *Lepidoglyphus destructor*	Sudden onset of lip and tongue swelling, throat tightness and shortness of breath, angioedema, wheezing, rhinorrhea, etc.	Anaphylaxis	Intramuscular epinephrine, antihistamines and steroids	[30–40]
Otic	China, Taiwan, Thailand	*Dermatophagoides pteronyssinus, Suidasia pontifica*	Severe itching, feeling of insects crawling in the ear	Otoscopic examination of mites	Eardrops containing triamcinolone, nystatin, neomycin and gramicidin	[64–66]
Vaginal	China	-	Vaginal itching, increased leukorrhea, low back pain, abdominal pain, and a sensation of abdomen falling	Identifying mites on leukorrhea smears	Metronidazole (3 times every day)	[67]

## Review

### Pulmonary acariasis

Pulmonary acariasis is a non-specific infestation of human lungs by free-living mites. In the 1930s, mites were observed in human sputum [4]. Subsequent experiments demonstrated that free-living mites can invade animal lungs and live in the respiratory tract. Indeed, Carter *et al.* [19] detected mites in sputum from 60.71% (17/28) of asthmatic patients. Interestingly, the authors thought the mites were derived from contamination of the test vessel because the detected mite species were present in dust samples from the same hospital and wards. After ruling out possible contamination, the authors repeatedly tested the sputum samples and obtained the same results. Among those 17 patients, one had severe asthma and had mites in his lungs for more than 7 months. Blood examination showed increased eosinophil numbers in all 17 patients. After treating patients with arsenic, the numbers of mites in their sputum samples increased, which showed that mites in the lungs were driven out, before the numbers of mites decreased and patients’ symptoms resolved [19].

Since that initial report, a number of other cases of pulmonary acariasis have been documented. In 1947, mites were detected in sputum of 3 out of 28 patients with tropical eosinophilia in East Africa [20]. In China, Gao *et al.* [21] reported for the first time that *Tyrophagus* and *Tarsonemus* were found in sputum of a bronchiectasis patient [21]. Ryu *et al.* [22] reported that a 23-year-old medical student showed a positive reaction on a skin test for *Paragonimus westermani,* and two *Tarsonemus floricolus* mites were subsequently found by sputum examination and identified morphologically. This was the first human infection with *Tarsonemus* reported in Korea. Further, in 2007 a new process for identifying mites in sputum was developed. Martínez-Girón R *et al.* [23] demonstrated that dust mites artificially introduced into sputa could be identified after sputa were liquified with bleach and the liquid sample was observed under the microscope. Their approach offers a time- and cost-saving tool for identifying dust mites in sputum, but the test is not commonly applied in the clinic because acariasis is not well recognized.

Van Woerden [24] proposed that asthma in house dust mite-sensitive patients may be caused by recurrent inhalation of live dust mites that are able to live for some time in the bronchioles of the lung. The mites may provide their own food source by excreting proteolytic enzymes—including the protein Der p 1, a major allergen—that free cells from the basement membrane to increase epithelial shedding. Shed respiratory epithelial cells can then be consumed by mites. However, this loss of respiratory epithelium can provoke sensitization to the dust mite proteins and other allergens, which later results in asthma symptoms. Indeed, the association between pulmonary acariasis and asthma has been demonstrated across studies.

Several papers published in Chinese reported on the etiology, pathology, diagnosis, and treatment of pulmonary acariasis. Their work suggested that the occurrence of pulmonary acariasis is related to occupation, with individuals who work in production, processing, and storage of food and herbs having higher risks of infection [25–27]. A few reports from China indicated that the infection rate and prevalence were highest in people aged 36-45 years, the second-highest rates were in 26-35 and 16-25 year-old people [28, 29]. Further, males were more commonly infected. However, these patterns may be influenced by occupational characteristics.

There are no specific clinical manifestations that point to pulmonary acariasis, beyond detection of mites in sputum. Patients with mild cases exhibit cold-like or bronchitis-like symptoms. Patients with severe cases often appear to have tuberculosis, pleurisy, or asthma, exhibiting symptoms such as cough, increased sputum volume, chest pain, shortness of breath, fatigue, fever, irritability, blood in sputum, and hemoptysis. A few patients have a severe cough in the morning and evening, accompanied by back pain, headache, dizziness, abdominal pain, and diarrhea. Except for increased eosinophil counts, no abnormalities are detected for red blood cells, hemoglobin, platelets, or liver function. A chest x-ray may show enhanced shadow in the hilar region and increased marking in the depth. Thus, pulmonary acariasis is often misdiagnosed as bronchitis, hilar lymphadenopathy, lung fluke disease, tuberculosis, or pleurisy [30–34]. Clinicians therefore proposed that, along with chest x-ray and blood counts, occupational history can be used for differential diagnosis. Treatment for pulmonary acariasis includes organoarsenic drugs like carbarsone and acetarsol, as well as drugs like hetrazan, thiodiphenylamine, emetine, and some antibiotics [30–34].

Chen *et al.* [35] reported typical nodular foci developed in the lungs of guinea pigs six days after five mite species, *Aeuroglyphus ovatus*, *Suidasia nesbitti*, *Dermatophagoides farinae*, *Tyrophagus putrescntae*, and *Acarus siro*, were injected into the tracheas. The foci occurred in all parts of the lobes, and were found to be yellow in fresh tissues and ranged from one to five millimeters in diameter. The pathological changes were associated mainly with bronchial and peribronchial lesions. Eosinophil infiltration was not observed in the lungs 20 days later. Multiple multinucleate giant cells grew and there was a striking formation of foreign body granuloma with the involvement of blood vessels. The parasitic mites were usually found in association with some arterioles. It was therefore demonstrated that guinea pigs offer an appropriate animal model for the study of pulmonary acariasis and that the five mites-species possess the same pathogenesis [35].

### Intestinal acariasis

Intestinal acariasis is caused by ingestion of mite-contaminated foods. This invasion of the human gastrointestinal tract can cause symptoms including diarrhea, abdominal pain, and burning sensation around the anus. The first case of intestinal acariasis was reported by Hinman & Kammeier [36], who detected *Tyrophagus longior* (Tyroglyphidae) in human intestine [36]. Three cases of intestinal acariasis were described in Spain; one of these was *Suidasia* mites [37]. Several cases of diarrhea were reportedly caused by *Carpoglyphus lactis*, presumed to have been transmitted through contaminated imported sugar [18, 38]. Other studies have identified cases of abdominal pain, diarrhea, fatigue, and pyohemofecia attributable to *Dermatophagoides farinae*, *D. pteronyssinus*, *Acarus siro*, *Tyrophagus putrescentiae*, *Carpoglyphus domesticus*, *Glycyphagus domesticus, G. privatus*, and *Tarsonemus granarius* [39–41]. Interestingly, an allergic intestinal acariasis syndrome has also been described [42]. Occasionally mite eggs, rather than (or in addition to) mites, have been found in stools [43, 44]. Indeed, Werneck *et al*. indicated that stool samples containing mite eggs, which were sometimes accompanied by adult mites, may often be misidentified as helminth ova, leading to erroneous treatment with far-reaching consequences [44].

Both direct fecal smear and a saturated salt solution floating method can be used for detection of mites in larva, adult, live, dead, or egg stage. Zhang *et al*. developed an avidin-biotin system enzyme-linked immunosorbent assay (ABC-ELISA) to aid diagnosis of intestinal acariasis [45]. Further, Li [46] suggested the broad-spectrum antiparasitic drug ivermectin as the first choice for treatment of human intestinal acariasis [46].

### Oral mite anaphylaxis (Pancake Syndrome)

In 1993, Erben *et al.* observed the first case of systemic anaphylaxis to mite-contaminated foods; the patient was treated with 0.3 mL of 1:1000 subcutaneous epinephrine, 75 mg oral diphenhydramine, and 40 mg of prednisone, and the symptoms gradually subsided over 90 minutes [47]. Later reports have described similar findings [48–54]. The name Pancake Syndrome derives from the commonality of patients being infected by mite-contaminated flour products. Indeed, a recent report by Takahashi *et al.* [55] summarized 36 cases with oral mite anaphylaxis in Japan. Of those, 34 had ingested okonomiyaki or takoyaki, Japanese pancakes prepared at home using mixes that were previously opened and stored for months at ambient temperature. Microscopic examination of those mixes revealed contamination with mites such as *Dermatophagoides farinae*, *Tyrophagus putrescentiae*, and *Dermatophagoides pteronyssinus* [55].

Matsumoto *et al.* indicated that two cases who developed systemic anaphylaxis shortly after eating food contaminated by a storage mite, *Tyrophagus putrescentiae*, were sensitive to storage mites but not to food allergens [49]. Similarly, Blanco *et al.* [56] investigated sixteen patients with respiratory allergies to dust mites and reported three of six food challenges with contaminated flours resulted in systemic reactions. Microscopic examination of four flours implicated in allergic reactions revealed a high degree of mite contamination: *Dermatophagoides farinae* in one case and *Thyreophagus entomophagus* in three cases [56]. Other reports identified *Blomia freemani* and *Thyreophagus entomophagus* in wheat flour as the source of anaphylaxis [51, 57]. Thus, ingestion of foods contaminated with mites may induce systemic anaphylactic reactions in patients with respiratory allergy to mites [56]. Systemic anaphylaxis can occur after the ingestion of heated or unheated mite-contaminated foods, and the most common symptoms are breathlessness, angioedema, wheezing, and rhinorrhea, beginning between 10 and 240 minutes after eating [50].

### Urinary acariasis

Urinary acariasis results from the presence of mites in the human urinary system. The first report of mites detected in human urine was published in 1938. These mites were identified as *Histiogaster* of the Tyroglyphidae family of storage mites [58]. Since then, other cases of urinary acariasis have been reported [59, 60]. One report described 7 cases with primary infection, pyelonephritis, and pyelocystitis resulting from numerous mites in the urinary sediment and, in some cases, also their eggs—some of which were motile, others were encrusted with salts [61]. One report indicated that, in a case with a few mite eggs in the urine, a six-legged mite larva emerged after the eggs were squashed on the slide. The authors suggest that the possibility of gut or bladder mite infection should be entertained only after repeated identification of mites in urine or stool samples from a symptomatic patient with no other cause for the symptoms and where the possibilities of contamination and spurious infection have been excluded [62].

Interestingly, a study in China of a sampling of individuals across different occupations indicated that 3.46% (69/1994) of urine samples contained adults, larvae, or eggs of mites [63]. Mites can damage urethral epithelia because they are good at digging. Furthermore, they can also invade loose connective tissue and small blood vessels in the urinary tract and cause localized ulcers. Undoubtedly, mites detected in urine under a microscope would contribute to diagnosis of this disease. Both chloroquine and metronidazole produce good responses for human urinary acariasis [46]. However, the pathogenesis of urinary acariasis remains uncertain.

### Otoacariasis

Less commonly, otoacariasis—or mite infestation in the ear—has been described. Mites were observed on the crusts taken from the radical mastoidectomy cavity and on the earwax from the external auditory canal of a female peasant in China; nearly all stages of the life cycle of the parasite were observed [64]. Similarly, a 70-year-old man in Taiwan presented with a 2-month history of pruritus and a sense of fullness in the right ear, and otoscopic examination revealed a number of mites and mite eggs in the right external auditory canal, which were identified as the house dust mite *Dermatophagoides pteronyssinus* [65]. This patient was treated with eardrops containing triamcinolone, nystatin, neomycin and gramicidin. Finally, in Thailand the external auditory canal of a 57-year-old woman was infested with >20 mites [66].

### Vaginal acariasis

In what is probably a similar mechanism to that for urinary acariasis, mites can parasitize the vagina. Chang *et al.* [67] described two cases of vaginal acariasis whose main symptoms were vaginal itching, increased leukorrhea, low back pain, abdominal pain, and a sensation of abdomen falling. Microscopic examination detected mites on leukorrhea smears [67]. Both patients were treated with metronidazole (3 times every day), which resulted in resolution of the infection.

## Conclusions

Domestic mites receive a lot of research and clinical attention because of their known allergenicity. However, their parasitic activities in humans are often overlooked. Given the existing reports, it seems likely that most cases of acariasis occur in more tropical climates and in people with occupational exposures to mites. The potential remains, though, that cases of acariasis go undiagnosed in other temperate climates. Considering the potential for misdiagnosis of acariasis, more effort should be devoted to understanding these infections, recognizing the populations most at-risk for infection, raising awareness among physicians for potential diagnosis, and identifying the best treatment options for each type of infection. This review highlights what is known about mites as human parasites, while also making clear that more work needs to be done to shed light on the occurrence of mite invasion and its associated symptoms.
